# The Association of Dietary Macronutrients with Lung Function in Healthy Adults Using the Ansan-Ansung Cohort Study

**DOI:** 10.3390/nu12092688

**Published:** 2020-09-03

**Authors:** Sang-Ah Lee, Pankaj Joshi, Yeonjin Kim, Daehee Kang, Woo Jin Kim

**Affiliations:** 1 Department of Preventive Medicine, Kangwon National University School of Medicine, 1, Kangwondaehak-gil, Chuncheon-si, Gangwon-do 24341, Korea; daccpankaj@gmail.com (P.J.); bbkeye1229@naver.com (Y.K.); 2 Division of Epidemiology, Vanderbilt University Medical Center, 1211 Medical Center Dr, Nashuville, TN 37232, USA; 3 Department of Preventive Medicine, Seoul National University College of Medicine, 103, Daehak-ro, Jongno-gu, Seoul 03080, Korea; dhkang@snu.ac.kr; 4 Department of Internal Medicine, School of Medicine, Kangwon National University, 1, Kangwondaehak-gil, Chuncheon-si, Gangwon-do 24341, Korea; 5 Environmental Health Center, 156, Baengnyeong-ro, Chuncheon-si, Gangwon-do 24289, Korea

**Keywords:** lung function of healthy population, difference of FEV_1_, macronutrient, longitudinal study, obese

## Abstract

This study is aimed to examine the association between macronutrient intake and lung function in healthy adults (*n* = 5880) using the Ansan-Ansung cohort study. To identify the index of lung function, we used the percentage difference of predicted Forced Expiratory Volume (%FEV_1_diff_) between baseline and follow-up. Based on the median %FEV_1_diff_, subjects were classified by two groups as “decreased vs. unchanged/improved”. The dietary macronutrients were estimated and validated using the food-frequency questionnaire. Multiple logistic regression models were used to evaluate the association after adjusting for confounders. Advanced analysis examined the association after stratifying by age and obesity. The average of %FEV_1_ is 114.1 and 112.5 at baseline and follow-up, respectively. The positive association of protein and fiber intake with lung function was observed in men. Low fat and high carbohydrate intake decreased the lung function in women only. After stratification by age, the association of protein, fat, and carbohydrate intake with lung function was observed in young men and old women only. Otherwise, the association of protein and fiber with lung function was influenced by abdominal obesity. In conclusion, the lung function was positively associated with high protein and fat intake, but was negatively associated with high carbohydrate intake, which could be influenced by age and obesity.

## 1. Introduction

Respiratory dysfunction is a life-threatening but treatable chronic disorder of the lung. Nutrition has been suggested as an important aspect in the care of respiratory disease. Malnutrition adversely affects lung function by diminishing respiratory muscle strength, altering ventilator capacity, and impairing immune function [1]. Even among men without COPD (Chronic Obstructive Pulmonary Disease), the lung function is associated with blood markers of nutritional status [2]. A prospective study of middle-aged men revealed a significant negative association between total energy intake and lung function. Regression coefficient suggested lung function (FEV_1_) was 48.8 mL (95% CI 21.4 to 76.3) lower for total energy intakes one standard deviation (597 kcal/day) apart [3]. Root et al. [4] summarized that macronutrient intake is correlated with lung function, and highlighted the positive association of animal protein with forced expiratory volume/forced vital capacity (FEV_1_/FVC (Forced Vital Capacity)). A report with COPD patents suggested that accurate evaluation of protein and energy requirements should be included in the goals of medical treatment of COPD patients [5].

Most Asians, including Koreans, consume relatively large amounts of carbohydrates (e.g., refined rice) as a staple food compared to Western countries but ingest low amounts of animal protein sources. Adults consume 62.2% and 14.9% of total energy intake from dietary carbohydrate and protein, respectively (https://knhanes.cdc.go.kr/knhanes/sub01/sub01_05.do#s5_03) as reported in the Korean Health Statistics 2016. Lee et al. reported that a low intake of protein is associated with airway obstruction in patients [6]. Otherwise, dietary fat is associated with impaired lung function in older men, which was attributed to induce innate immune activation and IL-6 (InterLeukin-6) release [7]. Cai et al., however, demonstrated experimentally that oral supplementation of high-fat and low-carbohydrate diets significantly increased lung function [8]. A study of Korean women reported that a high consumption of refined diets (high intake of natural carbohydrates) and low intake of fiber were associated with a decrease in lung function [9]. Otherwise, a high intake of dietary fiber was significantly associated with a higher percentage of individuals with normal lung function [10].

Although the effects of nutritional status on the respiratory system have been mainly focused on the prevention and prognosis of lung dysfunction, it is also very important to examine the association of macronutrients with pulmonary function in healthy adults. This is because an effect of nutrition on the respiratory disease incidence is accumulation over a long period of time. Therefore, this study is aimed to examine the association between macronutrient intake and lung function in healthy adults and to evaluate the association after stratification by age and both general and abdominal obesity.

## 2. Methods

### 2.1. Study Population

**Figure 1:**
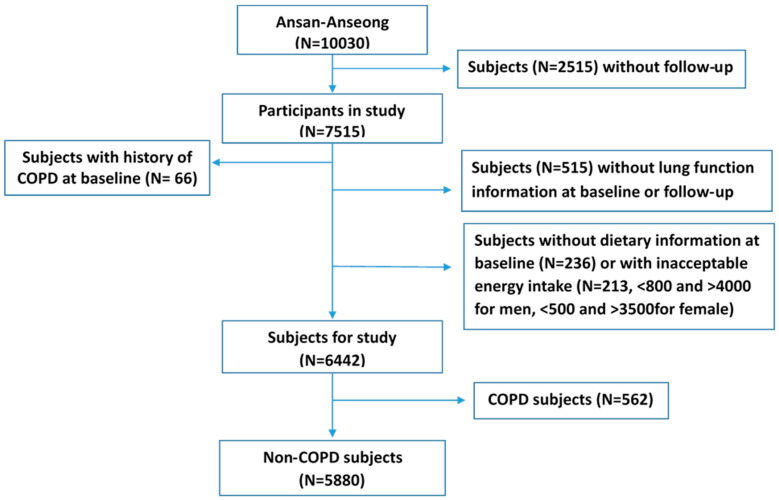
Flow diagram of analytical sample in current study using KoGES_Ansan and Ansung cohort. COPD: Chronic Obstructive Pulmonary Disease.

The study used the data from one of the community-based Korean Genome Epidemiology Study (KoGES) cohorts, namely the Ansan-Ansung cohort, and the detailed study design and procedures are reported elsewhere [11]. Spirometry was measured by three well-trained pulmonary technologists in concordance with the 1994 American Thoracic Society recommendation, using a spirometer (Vmax-229,Sensor-Medics, Yorba Linda, CA, USA) for all subjects. The predicted forced expiratory volume in one second (FEV1) were measured using a standardized method. To evaluate the lung function, we used the difference of FEV_1_ (_diff_ FEV_1_) from baseline (from May 2001 to February 2003) to follow-up (from February 2005 to November 2007) data. Prior informed consent was obtained from each participant during the recruitment and subsequent visits, which were approved by the Human Subjects Committee of Korea. The Korean National Institute of Health Institutional Review Committee approved the study procedures. Study procedures were in accordance with institutional guidelines and were approved by the Kangwon National University Hospital institutional review committee (IRB No. 2013-07-006) for the ethical approval. From the baseline (N = 10,030), subject with lost to follow-up (F/U) (N = 2515) couldn’t be included in the study. The distribution of ecological factors between eligible population and follow-up loss was presented in Supplementary Table S1. We excluded the participant who had a history of COPD at baseline (N = 66), without lung function information (FEV_1_) at baseline or F/U (N = 515), with unacceptable energy intake (less than 800 kcal or more than 4000 kcal for men and less than 500 kcal or more than 3500 kcal for women, N = 213), and without dietary information at baseline (N = 236). In addition, participants with COPD diagnosed at recruitment (*n* = 562) were excluded to eliminate a latent period bias. Finally, 5880 healthy subjects were included in this study (Figure 1). 

### 2.2. Covariates

Age was grouped by 5-year interval and education was categorized as two group, namely educated below than 12 years and more than 12 years. Household income per month was divided in two as below $2000 and more than $2000. Job was classified four groups (sedentary worker and labor, farmer, and housekeeper/other) from original eight job classification; (1) sedentary worker included officer, self-employment, and professional worker; (2) labor included sale officer and blue-colored worker; (3) farmer; and (4) housekeeper/other. Moreover, we separately made a farmer category because many subjects in the Ansung province among the cohort community engaged in agriculture (26.8%). BMI (Body Mass Index) was calculated by height (m) and weight (kg) (kg/m^2^) and categorized based on WHO (World Health Organization) criteria as less than <25, 25–29.9, and ≥30. Waist circumference was taken the Asian guidelines by National Cholesterol Education Program Adult Treatment Panel III (NCEP-ATP III) (men > 90, women > 80). We calculated the waist-to-hip ratio (WHR) using waist and hip circumference. For the lifestyle factors, cigarette smoking and alcohol drinking included three groups as never, former, and current. Participants were classified in terms of regular exercise based on the “yes” and “no” answer to the following question, “Do you currently engage in regular exercise strenuous enough to cause you to break into a sweat at least once per week?”.

### 2.3. Lung Function Measurement

To test a pulmonary function, Spirometry (VMAX2130 from Sensormedics Corporation, Yorba, CA, USA) was used to measure Forced expiratory volume (FEV_1,_ the amount of air that can force from lungs in one second) at baseline and follow-up visits. Spirometry was performed by trained technicians according to American Thoracic Society/European Respiratory Society guidelines [12]. Lung function was presented as percentage of predicted FEV_1_ (%FEV_1_), which calculated using measured and predicted FEV1 for each person (% FEV_1_ = base FEV_1_*100/predicted FEV_1_). To evaluate the lung function, we calculated the difference of %FEV1 (%FEV_1_diff_) between at baseline and follow-up. Based on the distribution of %FEV_1_diff_ (median = −1), we divided two groups for the lung function (decreased vs. unchanged/improved). The median value was involved in the unchanged/improved lung function group due to the maintenance of the %FEV_1_ despite the fact that aging means the lung function is relatively healthy.

### 2.4. Dietary Assessment

Dietary information was collected at baseline using validated and reproducible [13] semi-quantitative Food-Frequency Questionnaire (FFQ). Detailed information pertaining to dietary assessment is available in our previous report [13,14]. Briefly, FFQ consisted 103 food items and contained 9 serving categories for each item (never or seldom, once a month, 2–3 times a month, 1–2 times a week, 3–4 times a week, 5–6 times a week, once a day, twice a day, and 3 times or more every day) and the serving portion size (small, medium, and large). For the food items with different seasonal availability, we requested the participants to mark one on how long they ate among four categories: 3, 6, 9, and 12 months. The portion size was determined depending on the median value of each food determined from the 24-h recall data obtained from the Korean Health and Nutrition Examination Survey (KHANES). The portion size of each food item was classified as follows: small (0.5), medium (1.0), or large (1.5). For easy understanding of portion size, we provided pictures on serving size for food items on their own pages. Nutrient intake of each food item was converted based on the weight derived from the consumption frequency and the portion size in each food item. Daily nutrient intakes of everyone were the sum of the nutrient intake of each food item, which were calculated using DS24 (Human Nutrition Lab, Seoul National University & AI/DB Lab., Sookmyung Women’s University, 1996). The food composition table used in the two calculations was the 7th edition Food Composition Table of Korea (The Korean Nutrition Society, 2000).

### 2.5. Statistical Analysis

Ecological and lifestyle factors were presented the distribution using descriptive statistics and evaluated the association with lung function using logistic regression model after adjustment for age, education, income, marriage status, height, job, history of asthma, and history of tuberculosis as covariates. To examine the association between macronutrients and the lung function, a multiple logistic regression model was undertaken for the determination of odds ratios (OR) and the 95% confidence interval as increased interquartile range (IQR) after adjustment for age (continuous), education (<12 vs. ≥12 years), job, BMI (continuous), WHR (% of more than 0.9 and 0.85 for men and women respectively), smoking status (none, former, and current smoker), and energy intake (continuous), which were observed the association with the lung function in this population. If alpha = 0.05, total subject number = 5880, and estimated odds ratio = 1.1, the power for the association between decreased and unchanged/improved is 0.928 based on the logistic regression analysis. To evaluate the association of macronutrients as continuous variable, we had examined odds ratio as increased interquartile range of each macronutrient (OR_IQR_). We examined the advanced analyses after stratification by age, BMI and WHR (Waist-Hip Ratio). The age was classified two groups based on the median age (50 years old) of study population and was added the analysis for the elder (≥65 years old). For the general obese, we divided two groups as normal (BMI < 25) and overweight/obese (BMI ≥ 25). For the WHR, we divided two groups based on the cut point by NCEP-ATP III (cut-point = 0.90 and 0.85 for men and women, respectively) A *p*-value of <0.05 was considered as the level of significance. Two-sided probability tests were employed using SAS statistical software (version 9.2, SAS Institute Inc., Cary, NC, USA).

## 3. Results

Comparing the distribution of selected characteristics between the eligible population and those lost to follow-up, it was not statistically different to age, sex, BMI, and %FEV1 at baseline between eligible population and follow-up loss. Otherwise, those lost to follow-up were likely to less educated, lower income, not married, occupied as labor or housekeeper, and had lower abdominal obesity, compare to the eligible population. The average of %FEV_1_ is 114.1 ± 16.3 and 112.5 ± 15.6 (109.3 ± 14.5 and 107.6 ± 13.8 for men, 118.2 ± 16.6 and 116.6 ± 15.9 for women) at baseline and follow-up, respectively. The distribution of %FEV_1_ by lung function according to gender presented in Table 1. The lung function in men was negatively associated with age (elder), job (farmer), and abdominal obesity, and current smoking, but positively associated with low education (for women only) and general obesity (high BMI) (Table 1).

The percentage of energy from protein, fat, and carbohydrate is 13.8%, 15.7%, and 70.4% for men and 13.4%, 13.5%, and 73.1% for women, respectively. The distribution of energy percentage from each macronutrient was presented in Supplementary Table S2 The association between macronutrients intake and lung function was presented in Table 2 (ORs for IQR) and Supplementary Table S3 (ORs for quintile categories). The inverse association of protein (OR_IQR_ = 0.78) and fiber (OR_IQR_ = 0.85) intake with lung function was found in men. Fat (OR_IQR_ = 0.83) and carbohydrate (OR_IQR_ = 1.38) intake was associated with women’s lung function, but the total energy intake was not related to the lung function (Table 2).

To evaluate the modified effect of age (Table 3) and obese status (Table 4) on the association between macronutrients and lung function, we analyzed the advanced analysis after stratified by age, BMI, and WHR. Although lung function was not associated with energy intake in both men and women, an inverse association of protein and fat but positive association of carbohydrate with the decreased lung function was observed in young men (<50 years old, OR_IQR_ = 0.72, 0.80 and 1.47 for protein, fat and carbohydrate, in order) and elderly women (both ≥50 and ≥65 years old, OR_IQR_ = 0.74, 0.56 and 3.21 for protein, fat and carbohydrate, in order) (Table 3). Otherwise, fiber intake was shown in the opposite direction as age group. High fiber intake was inversely associated with unchanged/improved lung function in younger women (OR_IQR_ = 1.44) but was positively associated with the lung function in older men (OR_IQR_ = 0.78).

In advanced analysis after stratified by BMI, no association of each macronutrients with lung function with exception of fat intake (OR_IQR_ = 0.78 in women with normal weight). Otherwise, after stratified by waist-to-hip ratio, we observed the inverse association of protein (OR_IQR_ = 0.67) and fiber (OR_IQR_ = 0.76) intake with decreased lung function in men with normal waist-hip ratio. Among women with normal waist-hip ratio, the inverse association of total energy intake (OR_IQR_ = 0.85) with decreased lung function. Furthermore, the decreased lung function was associated inversely with fat (OR_IQR_ = 0.81), but associated positively with carbohydrate (OR_IQR_ = 1.37) intake among women with abdominal obesity.

## 4. Discussion

This study suggested that protein and fat intake was inversely associated with lung function decline, but carbohydrate intake was positively associated. The association was influenced by age and both general and abdominal obese. The inverse association of protein and fat was shown in men with below median age, but in women with above median age. Additionally, low fat intake was decreased the lung function in only women with abdominal obesity. Lung function was associated with high carbohydrate intake in women, predominantly older women (both >50 and >65 years old) and was declined in women with normal BMI or abdominal obese as carbohydrate intake increased. The association of fiber intake with lung function decline is inversely associated in men with above median age or with normal WHR, but positively associated with in the women with below median age.

Inverse correlation with unchanged/improved lung function (%FEV_1_diff_) with age was observed only in men. The working in farm was associated with the decreased lung function which could be explained to exposure to inorganic and organic dust, supported by other studies [15,16]. We found the negative correlation of BMI with %FEV_1_diff_ consistent with a recent report [17]; although the CARDIA study (Coronary Artery Risk Development In young Adults study) suggested that the obesity epidemic threatens the lung health of the general population [18]. Several studies have reported that abdominal obesity is related to decreased pulmonary function, consistent to ours result [19]. A recent clinical study using directly measured visceral adipose tissue and subcutaneous adipose tissue through magnetic resonance imaging, dual-energy radiography absorptiometry, and computed tomography (CT) reported that decreasing abdominal visceral obesity could increase lung function despite ageing [20]. The epidemiologic and clinical evidence over the past 50 years suggested the biological plausibility of a link between cigarette smoking and adverse respiratory outcomes [21,22]. A recent report using the South Carolina Behavioral Risk Factor Surveillance System (BRFSS) discusses the adverse effects of smoking duration and number of pack-years on lung function [23]. In this study population, the association of smoking with lung function was found in men only.

Although Butland et al. found an inverse association between lung function and energy intake [3], the total energy intake was not significantly associated with increase in lung function in our study, with the exception of the protective effect in females with normal BMI. Therefore, the role of energy intake on the lung function is controversial and needs further investigation. In the present study, we observed a significant association between higher protein intake and better lung function in both healthy men and women supported by Beasley’s results [24], although gender differences play a vital role in relation to lung function [25]. After stratified by age group, the association was predominantly observed in women with above median age, even in elderly women, similar to the result from a previous report [26]. This finding could be supported by the fact that the protein needs of elderly females could be higher [27] and the high protein intake leads to decrease health problems in elderly women [28].

The effect of fat intake on the decreased lung function was controversial and inconsistent because of the bi-directionally mechanical role on the pulmonary system. The harmful effect of fat was explained by bronchial hyper-responsiveness [29], incidence of asthma [30], a higher risk for COPD [31] and innate immune activation [7], which can lead to a systemic inflammatory response. The systemic inflammatory response includes the high level of circulating pro-inflammatory mediators. Otherwise, the beneficial effect of fat on the lung function was reported with many possible mechanisms; (1) omega-3 fatty acids protect the lung function by inhibiting the production of prostaglandin E2, thereby preventing allergic sensitization [32], (2) the intake of omega-6 fatty acids associated with the decreased risk of chronic nonspecific lung disease [32], (3) fat helps in digestion, absorption, and transportation of fat-soluble antioxidant vitamins; vitamin A, D, E and K [33], which could improve lung function [14,34]. and (4) fat metabolism generates less CO_2_ which has a lower respiratory quotient [35]. Our study suggested the protective effect of fat intake on lung function of relatively young men, elderly women, and abdominal obese women, contrary to one of the published results [7] which suggested a reduced lung function in older men as increased proportion of fat in the diet. Although our results suggest an inverse association of fat intake with lung function decline, it is necessary to advanced analysis considering the several limitations, such as the lack information of fat intake including animal vs. plant fat, the composition of trans-fat, saturated vs. polyunsaturated fatty acid, *w*-3 vs. *w*-6, etc. Therefore, advanced analyses considering these factors are needed to explain the protective effect on the lung function.

In the healthy population, carbohydrate metabolism increases CO_2_ production and respiratory quotient [35]. Although individuals with normal lungs eliminate excess loads of CO_2_ easily [36], the long-term effects suggest deterioration in lung function, especially in the elderly. Therefore, a lower carbohydrate diet might prevent or increase respiratory health [37]. The possibility that altered respiratory variables after ingestion of carbohydrate were nonspecific and unrelated remote for the following reasons. The metabolic and respiratory responses to ingestion of carbohydrate confirm qualitatively and quantitatively to previous observations and predictions [38,39,40,41,42]. Consistent with previous reports [31,43,44,45], a carbohydrate-rich diet is negatively associated with predicted FEV_1_, consistent with our result as well as another report with Korean women [46].

Several studies had reported an inverse association of dietary intake of fiber and lung function decline [10,47]; we found the association in older men only (more than 50 years old). For younger women, otherwise, high fiber intake was positively associated with the decreased lung function in this study population. To illustrate the positive association between high fiber intake and lung function decline in women, it is valuable to consider the source of dietary fiber in Korean diet. The food groups that contributed most to dietary fiber intake were (in descending order) cereals, vegetables, seasoning, and fruits in Korean and the fiber-containing food items consumed most were cabbage- kimchi, cooked rice, instant noodles, and cabbage [48]. Unfortunately, the difference between sex and age in Korean dietary fiber sources has not been studied yet. Therefore, further studies are needed to explain the relationship of dietary fiber to lung function.

Our study has several limitations. First, although it is acceptable to use FVE_1_ for the assessment of the pulmonary function in the epidemiology study, the association between macronutrients intake and the lung function could be underestimated due to the non-differential misclassification of the lung function. In addition, the association should be illustrated with consideration of the different follow-up time among the participants and annual change in lung function as aging. Therefore it is difficult to accurately adjust the effect of pulmonary function decline as increasing age. Second, we used FFQ that depended on the subjects’ memories of dietary intake and possible difficulty in accurate recall of frequency and food portion size. To overcome this limitation, we used a closed format and the ability to exercise different options based on food illustrations to facilitate easier recall. Besides, dietary information was collected only at baseline and changes during follow up period were not taken into account. Third, the findings cannot be generalized to populations other than Korean, especially in the Western countries with high-protein diets, because most of our participants were Korean. Possibly, our result could apply to Asia countries where the primary food are carbohydrate-rich (especially, starch-based refined rice). Forth, we had considered only macronutrient intake to evaluate the effect on the lung function. However, an advanced analysis is required considering the quality of the diet, such as plant vs. animal protein, sugar vs. complex carbohydrate, the very low fiber in the diet. Fifth, smokers at baseline may have quit smoking between the lung function measurements, which may have had impact on lung function. In addition, we couldn’t consider the change of the general and abdominal obesity status during follow-up. Finally, the high proportion of follow-up loss could be affected by selection bias, although we tried to adjust for education, married status, job abdominal obesity, which were observed a different distribution between the eligible population and follow-up loss.

Aside from these limitations, our study has several strengths. First, the use of data from a large prospective cohort allows the possibility of a temporal relationship, although causality cannot be assessed unless some strong assumptions are made (e.g., missing at random conditional on observed variables). Second, we included well-known confounding factors in the analysis such as age, sex, marital status, BMI, WHR, history of asthma and tuberculosis, and cigarette smoking, because of the large sample size. Third, FFQ was developed based on nationwide dietary data, and hence, the use of a validated FFQ strengthened the reproducibility of our results. Fourth, to avoid the latent period bias and to preclude the bias related with altered lifestyle factors, because of inconvenience lung function without a diagnosis of respiratory disease, we excluded subjects who were diagnosed with COPD at recruitment. Fifth, this report examined advanced analytics to evaluate the advanced analysis after stratification by age and both general and abdominal obese status on the association between macronutrient intake and the lung function decline.

## 5. Conclusions

In conclusion, this study suggested a positive association of protein and fat intake but a negative association of carbohydrate intake with the lung function in the healthy population. Furthermore, the association between macronutrients and lung function could be attenuated by age and obese status.

## Figures and Tables

**Table 1:** The distribution of difference of %FEV_1_ (lung function) and the association with general and lifestyle factors according to gender.

	Men				Women			
	Unchanged/Improved	Decreased	Crude OR	Adj OR	Unchanged/Improved	Decreased	Crude OR	Adj OR
	(*n* = 1432)	(*n* = 1270)	95% CI	95% CI	(*n* = 1583)	(*n* = 1595)	95% CI	95% CI
% FEV_1_ at baseline	105.7 ± 13.0	113.4 ± 15.0			113.9 ± 15.9	122.6 ± 16.3		
% FEV_1_ at follow-up	110.5 ± 12.9	104.3 ± 13.9			120.3 ± 15.6	113.0 ± 15.3		
**Ecological Factors**								
Age (age, mean ± SD)	49.1 ± 7.67	51.4 ± 8.33	1.04 (1.03–1.05)	1.02 (1.00–1.03)	51.6 ± 8.81	52.3 ± 8.64	1.01 (1.00–1.02)	1.00 (0.98–1.01)
Education (>12 years, %)	69.7	59.5	0.63 (0.54–0.74)	0.94 (0.76–1.16)	37.9	29.7	0.70 (0.60–0.81)	0.81 (0.66–0.99)
Income (≥2000$/M, %)	54.0	41.9	0.62 (0.53–0.72)	0.98 (0.80–1.20)	34.0	28.2	0.76 (0.66–0.89)	0.98 (0.81–1.19)
Marriage status (No)	3.3	4.4	1.36 (0.92–2.02)	1.14 (0.71–1.81)	13.8	13.7	0.99 (0.81–1.21)	1.04 (0.81–1.34)
Job								
Sedentary worker	47.2	35.7	Reference	Reference	11.9	8.8	Reference	Reference
Labor	9.7	6.8	0.97 (0.73–1.22)	1.06 (0.78–1.44)	6.3	6.9	0.59 (0.29–1.21)	0.72 (0.33–1.58)
Farmer	18.6	37.6	2.54 (2.10–3.09)	2.16 (1.66–2.80)	17.7	26.5	1.64 (1.38–1.95)	1.49 (1.18–1.89)
Housekeeper/Others	24.5	20.0	0.91 (0.75–1.11)	0.92 (0.74–1.15)	64.1	57.8	0.86 (0.66–1.12)	0.78 (0.57–1.05)
BMI (kg/m^2^)	24.7 ± 2.72	24.2 ± 2.74	0.93 (0.91–0.96)	0.94 (0.91–0.97)	25.0 ± 3.24	24.9 ± 3.22	0.99 (0.97–1.02)	0.98 (0.95–1.00)
Abdominal obesity * (%)	37.8	50.1	1.65 (1.41–1.92)	1.32 (1.07–1.63)	53.2	63.5	1.53 (1.33–1.76)	1.33 (1.09–1.61)
His of Asthma (yes)	1.12	0.79	0.70 (0.32–1.55)	1.05 (1.07–1.63)	1.83	2.26	1.24 (0.75–2.03)	1.18 (0.65–1.22)
His of Tuberculosis (yes)	4.96	6.30	1.29 (0.93–1.79)	1.23 (0.85–1.79)	3.85	3.20	0.83 (0.57–1.20)	0.77 (0.50–1.19)
**Lifestyle Factors**								
Cigarette smoking								
Never	23.0	20.3	Reference	Reference	96.0	95.7	Reference	Reference
Former	35.8	30.6	0.91 (0.75–1.11)	0.91 (0.73–1.14)	1.5	1.0	0.65 (0.34–1.25)	0.54 (0.25–1.17)
Current	41.3	49.1	1.28 (1.06–1.55)	1.28 (1.03–1.59)	2.5	3.4	1.35 (0.80–2.27)	1.23 (0.68–2.20)
Alcohol drinking								
Never	17.2	18.6	Reference	Reference	70.9	71.7	Reference	Reference
Former	9.0	9.4	0.99 (0.79–1.24)	1.11 (0.91–1.35)	2.7	2.6	1.05 (0.89–1.22)	0.95 (0.79–1.15)
Current	73.9	72.0	1.00 (0.73–1.37)	1.08 (0.83–1.40)	26.5	25.8	1.00 (0.64–1.53)	0.91 (0.54–1.53)
Exercise (yes)	33.3	31.5	0.92 (0.78–1.08)	1.03 (0.86–1.25)	28.1	25.0	0.85 (0.73–1.00)	0.96 (0.80–1.16)

Unchanged and improved: unchanged or inclined lung function, more than median (−1) of the difference between %FEV_1_ at baseline and follow-up. Decreased: under the median (−1) of the difference between %FEV1 at baseline and follow-up. % FEV1: Percentage of the annual change in lung function due to aging predicted FEV_1_. Abdominal obesity *: waist-to-hip ratio ≥ 0.9 or ≥ 0.85 for men and women, respectively. Adj OR: OR after adjusted for age, education, income, marriage status, BMI, waist-to hip ratio, job, history of asthma, and history of tuberculosis.

**Table 2:** The association between macronutrient intake and the lung function among healthy population.

	Men			Women		
	Unchanged/Improved	Decreased	OR_IQR_ (95% CI)	Unchanged/Improved	Decreased	OR_IQR_ (95% CI)
	(*n* = 1432)	(*n* = 1270)		(N = 1583)	(N = 1595)	
Energy (kcal/day)	1934 *	1901	0.98 (0.88–1.09)	1776	1739	0.96 (0.88–1.05)
Protein (g/day)	67.3	63.8	0.78 (0.64–0.96)	59.7	56.2	0.87 (0.72–1.06)
Fat (g/day)	34.2	31.3	0.97 (0.83–1.13)	26.0	23.5	0.83 (0.71–0.96)
Carbohydrate (g/day)	331.2	330.3	1.14 (0.89–1.46)	318.2	313.1	1.38 (1.09–1.75)
Fiber (g/day)	6.44	6.19	0.85 (0.72–0.99)	6.43	6.41	1.15 (0.99–1.34)

*: median of each macronutrients. OR_IQR_: OR as increased IQR (Q3–Q1) after adjusted for age, education, BMI, waist-to-hip ratio, job, smoking status and total energy intake; Unchanged and improved: more than median (−1) of the difference between %FEV1 at baseline and follow-up. Decreased: under the median (−1) of the difference between %FEV1 at baseline and follow-up.

**Table 3:** The association between macronutrient intake and the lung function by median age (50 years old) and among elderly participants.

	Men			Women		
	Unchanged/Improved	Decreased	OR_IQR_ (95% CI)	Unchanged/Improved	Decreased	OR_IQR_ (95% CI)
Below Median (Unchanged or improved = 1688, Decreased = 1376)						
Energy (kcal/day)	1945 *	1908	0.92 (0.79–1.06)	1821	1783	0.96 (0.84–1.09)
Protein (g/day)	68.8	65.2	0.72 (0.54–0.95)	62.5	59.9	1.01 (0.77–1.34)
Fat (g/day)	35.9	32.7	0.80 (0.64–1.00)	30.0	28.2	0.83 (0.68–1.03)
Carbohydrate (g/day)	331.1	329.0	1.47 (1.04–2.08)	322.9	315.4	1.33 (0.94–1.88)
Fiber (g/day)	6.36	6.07	0.93 (0.74–1.16)	6.26	6.45	1.44 (1.15–1.80)
Above Median (Unchanged or improved = 1063, Decreased = 1185)						
Energy (kcal/day)	1915	1888	1.14 (0.90–1.21)	1721	1683	0.96 (0.85–1.08)
Protein (g/day)	64.9	62.7	0.86 (0.63–1.17)	56.3	52.9	0.74 (0.56–0.97)
Fat (g/day)	30.3	29.4	1.17 (0.93–1.47)	22.1	20.3	0.81 (0.65–0.99)
Carbohydrate (g/day)	331.7	332.0	0.87 (0.60–1.25)	314.7	310.2	1.47 (1.05–2.05)
Fiber (g/day)	6.60	6.40	0.78 (0.62–0.98)	6.64	6.36	0.98 (0.80–1.19)
Elder (≥65 years old, Unchanged or improved = 264, Decreased = 304)						
Energy (kcal/day)	1790	1901	1.03 (0.74–1.45)	1699	1584	0.86 (0.67–1.10)
Protein (g/day)	59.2	57.7	0.90 (0.43–1.90)	53.8	47.2	0.48 (0.24–0.96)
Fat (g/day)	25.4	26.4	1.69 (0.97–2.92)	19.0	16.6	0.56 (0.31–0.98)
Carbohydrate (g/day)	327.6	341.9	0.62 (0.26–1.50)	316.2	302.5	3.12 (1.21–8.02)
Fiber (g/day)	6.78	6.14	0.88 (0.51–1.53)	6.46	6.08	1.05 (0.70–1.57)

*: median of each macronutrients. OR_IQR_: OR as increased IQR (Q3–Q1) after adjusted for age, education, BMI, waist-to-hip ratio, job, smoking status and total energy intake; Unchanged or improved: more than median (−1) of the difference between %FEV1 at baseline and follow-up. Decreased: under the median (−1) of the difference between %FEV1 at baseline and follow-up.

**Table 4:** The association between macronutrient intake and the lung function by general (BMI) and abdominal (WHR) obesity.

	Men			Women		
	Unchanged/Improved	Decreased	OR_IQR_ (95% CI)	Unchanged/Improved	Decreased	OR_IQR_ (95% CI)
**General Obesity**						
BMI < 25 (kg/m^2^)						
Energy (kcal/day)	1914 *	1875	0.98 (0.86–1.11)	1775	1736	0.91 (0.82–1.01)
Protein (g/day)	65.9	62.4	0.80 (0.61–1.03)	59.7	55.9	0.84 (0.67–1.07)
Fat (g/day)	33.1	30.6	1.02 (0.84–1.23)	26.0	23.4	0.79 (0.66–0.95)
Carbohydrate (g/day)	330.2	326.9	1.05 (0.77–1.42)	319.2	311.2	1.44 (1.08–1.93)
Fiber (g/day)	6.42	6.12	0.82 (0.67–1.01)	6.51	6.29	1.12 (0.93–1.34)
BMI ≥ 25 (kg/m^2^)						
Energy (kcal/day)	1955	1970	0.98 (0.82–1.18)	1777	1759	1.07 (0.92–1.25)
Protein (g/day)	69.8	67.2	0.74 (0.53–1.03)	59.6	57.1	0.91 (0.66–1.27)
Fat (g/day)	35.1	32.9	0.86 (0.65–1.14)	25.8	24.0	0.89 (0.68–1.16)
Carbohydrate (g/day)	332.9	336.9	1.35 (0.88–2.09)	314.8	318.4	1.28 (0.84–1.96)
Fiber (g/day)	6.51	6.49	0.89 (0.68–1.17)	4.82	6.69	1.24 (0.96–1.60)
**Abdominal Obesity**						
WHR (<0.9 and <0.8 for men and women, respectively)						
Energy (kcal/day)	1937	1877	0.89 (0.77–1.03)	1805	1774	0.85 (0.73–0.98)
Protein (g/day)	68.3	63.6	0.67 (0.49–0.90)	62.5	59.7	0.86 (0.61–1.21)
Fat (g/day)	35.4	31.5	0.94 (0.75–1.18)	30.0	27.8	0.89 (0.70–1.14)
Carbohydrate (g/day)	327.7	325.6	1.23 (0.85–1.77)	318.3	311.9	1.28 (0.86–1.92)
Fiber (g/day)	6.44	6.12	0.76 (0.59–0.98)	6.41	6.21	1.23 (0.95–1.60)
WHR (≥0.9 and ≥0.8 for men and women, respectively)						
Energy (kcal/day)	1927	1933	1.07 (0.92–1.25)	1742	1719	1.02 (0.92–1.14)
Protein (g/day)	65.4	64.2	0.90 (0.68–1.19)	55.5	54.1	0.90 (0.71–1.14)
Fat (g/day)	32.4	31.2	1.00 (0.81–1.25)	22.3	21.5	0.81 (0.68–0.98)
Carbohydrate (g/day)	335.9	337.4	1.04 (0.73–1.47)	317.8	313.6	1.37 (1.02–1.85)
Fiber (g/day)	6.46	6.32	0.90 (0.73–1.12)	6.42	6.54	1.12 (0.93–1.33)

*: median of each macronutrients. Bold: statistically significant association (*p* < 0.05). OR_IQR_: OR as increased IQR (Q3–Q1) after adjusted for age, education, BMI, waist-to-hip ratio, job, smoking status and total energy intake; The adjustment for BMI and WHR in advanced analysis after stratified by BMI and WHR, we had taken each confounder as crossing each other. Unchanged/improved: more than median (−1) of the difference between %FEV1 at baseline and follow-up. Deceased: under the median (−1) of the difference between %FEV1 at baseline and follow-up.

## Supplementary Materials

The following are available online at https://www.mdpi.com/2072-6643/12/9/2688/s1, Table S1: Distribution of ecological factors between eligible study population and follow-up loss, Table S2: Distribution of macronutrients intake and percentage of total energy intake by age group, BMI, and WHR, Table S3: The association between macronutrient intake and lung function among healthy population using five categorical analysis.
