# Supplementary material for: The Association of Dietary Macronutrients with Lung Function in Healthy Adults Using the Ansan-Ansung Cohort Study
Source: Nutrients. 2020 Sep 3;12(9):2688. doi: 10.3390/nu12092688 (PMC7551845; doi:10.3390/nu12092688)
Supplement: Supplementary file 1 [file nutrients-12-02688-s001.pdf]

Table S1. Distribution of ecological factors between eligible study population and follow-up loss

	Eligible population (n=7515)	Follow-up loss (n=2515)	P value
% FEV_1 at baseline	112.2±17.6	110.8±18.2	0.3675
Age (age, mean±SD)	52.2±8.83	52.4±9.25	0.5849
Sex (female, %)	52.3	53.5	0.2874
Education (>12 years, %)	43.9	40.0	0.0004
Income (≥2000$/M, %)	34.5	32.6	0.0517
Marriage status (No)	10.2	13.4	<0.0001
Job			
Sedentary worker	22.4	22.0	0.0060
Labor	6.6	7.33	
Farmer	29.5	21.9	
Housekeeper/Others	41.5	48.9	
BMI (kg/m^2)	24.6±3.07	24.5±3.13	0.1383
Abdominal obesity* (%)	53.9	51.0	0.0104
His of Asthma (yes)	2.25	2.03	0.2527
His of Tuberculosis (yes)	4.88	4.81	0.2472

Abdominal obesity*: waist-to-hip ratio≥0.9 or ≥0.80 for men and women, respectively.

Table S2. Distribution of macronutrients intake and percentage of total energy intake by age group, BMI, and WHR

	Men (% of total energy intake)				Women (% of total energy intake)			
	Protein	Fat	Carbohydrate	Fiber*	Protein	Fat	Carbohydrate	Fiber*
Age								
<50.0	70.1 (13.9)	37.4 (16.6)	341 (70.0)	6.62 (1.46)	64.5 (13.7)	31.8 (14.7)	327 (71.0)	6.78 (1.49)
50-70	66.4 (14.0)	32.7 (15.1)	347 (71.0)	6.94 (1.58)	57.5 (13.0)	24.1 (12.0)	328 (74.7)	7.00 (1.65)
elder (>=65)	62.9 (13.5)	29.6 (13.8)	350 (72.6)	6.89 (1.61)	52.7 (12.3)	20.3 (10.4)	328 (77.2)	6.87 (1.68)
BMI								
<25	66.8 (13.7)	34.4 (15.3)	339 (70.8)	6.63 (1.47)	60.1 (14.0)	28.2 (16.1)	321 (69.9)	6.81 (1.48)
≥25	71.0 (13.4)	36.8 (13.8)	349 (72.8)	6.93 (1.61)	61.0 (13.3)	27.3 (15.0)	332 (73.6)	6.99 (1.63)
WHR								
Normal	69.0 (14.0)	36.2 (16.1)	340 (69.4)	6.65 (1.44)	63.4 (13.5)	31.0 (15.3)	319 (71.8)	6.66 (1.52)
AO	68.1 (13.9)	34.5 (15.1)	348 (71.0)	6.89 (1.57)	58.9 (12.9)	25.5 (12.3)	335 (75.1)	7.06 (1.66)

*, % of total macronutrients intake

Table S3. The association between macronutrient intake and lung function among healthy population using five categorical analysis

	Men			Women		
	Unchanged /improved	Decreased	OR_{IQR} (95%CI)	Unchanged /improved	Decreased	Adj OR (95%CI)
Energy (kcal/day)						
Q1 (≤1474)			Reference			Reference
Q2 (1475-1722)	18.37	18.90	0.92 (0.69-1.22)	21.35	21.00	0.90 (0.73-1.11)
Q3 (1723-1960)	22.70	21.26	0.91 (0.69-1.20)	18.38	18.18	0.93 (0.75-1.15)
Q4 (1961-2297)	25.14	21.65	0.86 (0.65-1.13)	18.45	15.61	0.78 (0.62-0.97)
Q5 (≥2298)	22.28	23.62	0.92 (0.70-1.21)	17.56	17.49	0.88 (0.70-1.09)
			P_{trend}= 0.597			P_{trend}= 0.418
Protein (g/day)						
Q1 (≤45.29)	11.38	14.88	Reference	23.63	28.21	Reference
Q2 (45.30-56.15)	17.74	19.61	0.96 (0.72-1.30)	20.72	21.63	0.89 (0.71-1.11)
Q3 (56.16-67.14)	20.39	21.81	0.95 (0.69-1.32)	19.27	18.93	0.84 (0.65-1.10)
Q4 (67.15-80.96)	24.37	20.71	0.73 (0.50-1.05)	19.33	16.18	**0.68 (0.50-0.94)**
Q5 (≥80.97)	26.12	22.99	0.67 (0.43-1.05)	17.06	15.05	0.68 (0.46-1.02)
			**P_{trend}= 0.029**			**P_{trend}= 0.047**
Fat (g/day)						
Q1 (≤17.48)	10.41	13.15	Reference	24.13	29.97	Reference
Q2 (17.49-24.70)	15.50	18.50	1.06 (0.79-1.45)	22.43	22.82	0.87 (0.72-1.10)
Q3 (24.71-32.61)	19.76	21.97	1.11 (0.82-1.51)	19.96	18.68	0.82 (0.65-1.04)
Q4 (32.62-43.42)	25.77	21.65	0.87 (0.63-1.22)	17.56	15.92	0.82 (0.63-1.08)
Q5 (≥43.43)	28.56	24.72	0.84 (0.58-1.23)	15.92	12.60	**0.69 (0.50-0.95)**
			P_{trend}= 0.121			**P_{trend}= 0.032**
Carbohydrate (g/day)						
Q1 (≤270.4)	12.85	15.28	Reference	24.57	25.64	Reference
Q2 (270.5-306.0)	21.58	19.13	0.85 (0.64-1.14)	18.89	20.38	1.15 (0.92-1.45)
Q3 (306.1-341.8)	21.93	22.36	1.07 (0.77-1.48)	19.27	17.12	1.11 (0.84-1.46)
Q4 (341.9-398.5)	23.67	21.26	0.95 (0.64-1.41)	19.08	16.61	1.19 (0.84-1.39)
Q5 (≥398.6)	19.97	21.97	1.02 (0.60-1.72)	18.19	20.25	**1.75 (1.08-2.86)**
			P_{trend}= 0.835			**P_{trend}= 0.026**
Fiber (g)						
Q1 (≤4.41)	18.65	20.16	Reference	19.90	21.19	Reference
Q2 (4.42-5.74)	19.34	21.89	1.12 (0.87-1.43)	19.14	19.94	1.05 (0.83-1.31)
Q3 (5.74-7.06)	22.14	20.87	0.96 (0.74-1.24)	20.28	17.12	0.89 (0.70-1.13)
Q4 (7.06-8.91)	21.65	18.58	0.88 (0.67-1.16)	20.03	19.62	1.05 (0.82-1.35)
Q5 (≥8.92)	18.23	18.50	0.83 (0.61-1.12)	20.66	22.13	1.14 (0.86-1.51)
			P_{trend} =0.080			P_{trend}= 0.345

OR: OR after adjusted for age, BMI, waist-to-hip ratio, job, smoking status and total energy intake
Unchanged/improved: more than median (-1) of the difference between %FEV1 at baseline and follow-up
Deceased: under the median (-1) of the difference between %FEV1 at baseline and follow-up
